# Efficacy of canakinumab in mild or severe COVID‐19 pneumonia

**DOI:** 10.1002/iid3.400

**Published:** 2021-01-19

**Authors:** Falasca Katia, Di Penta Myriam, Claudio Ucciferri, Antonio Auricchio, Marta Di Nicola, Michele Marchioni, Celletti Eleonora, Sabatini Emanuela, Francesco Cipollone, Jacopo Vecchiet

**Affiliations:** ^1^ Department of Medicine and Science of Aging, Clinic of Infectious Diseases University “G. d'Annunzio” Chieti‐Pescara Chieti Italy; ^2^ Department of Medicine and Aging Sciences, Internal Medicine “G. D'Annunzio” University Chieti Italy; ^3^ Department of Medicine and Health Sciences University of Molise Campobasso Italy; ^4^ Department of Medical, Oral and Biotechnological Sciences, Laboratory of Biostatistics G. d'Annunzio University of Chieti Chieti Italy

**Keywords:** IL1‐β, monoclonal antibody, safety, SARS COV2, therapy

## Abstract

**Background:**

Clinicians all around the world are currently experiencing a pandemic caused by severe acute respiratory syndrome coronavirus 2 (SARS‐CoV‐2). Several therapeutic strategies have been used until now but, to date, there is no specific therapy to treat SARS‐CoV‐2 infection. In this study, we used canakinumab, a human monoclonal antibody targeting interleukin‐1 beta to improve respiratory function and laboratory parameters compared with standard therapy (hydroxycloroquine plus lopinavir/ritonavir).

**Methods:**

We enrolled 34 patients with mild or severe non intensive care unit (ICU) coronavirus disease 2019 (COVID‐19): 17 patients treated with standard therapy and 17 patients treated with a subcutaneous single dose of canakinumab 300 mg. We collected data about oxygen supports and laboratory parameters such as inflammation indices and hemogasanalysis. We compared the data collected before the administration of canakinumab (T0), 3 days after T0 (T1) and 7 days after T0 (T2) with the same data from patients taking the standard therapy.

**Results:**

We observed a reduction in inflammation indices and a significant and rapid increase in P/F ratio in canakinumab group, with improvement of 60.3% after the administration. We reported a significant reduction in oxygen flow in patients treated with canakinumab (−28.6% at T1 vs. T0 and −40.0% at T2 vs. T1). Conversely, the standard group increased the supply of high oxygen at T1 versus T0 (+66.7%), but reduced oxygen flows at T2 versus T1 (−40.0%).

**Conclusion:**

In hospitalized adult patients with mild or severe non ICU COVID‐19, canakinumab could be a valid therapeutic option. Canakinumab therapy causes rapid and long‐lasting improvement in oxygenation levels in the absence of any severe adverse events.

## Graphical abstract

In hospitalized adult patients with mild or severe non intensive care unit coronavirus disease 2019, canakinumab could be a valid therapeutic option. Canakinumab therapy causes rapid and long‐lasting improvement in oxygenation levels in the absence of any severe adverse events.

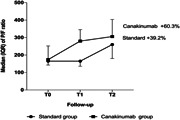

## 1 INTRODUCTION

A new challenge has recently emerged for the healthcare community across the world: the infection caused by a novel coronavirus, officially known as severe acute respiratory syndrome coronavirus 2 (SARS‐CoV‐2), responsible of a clinical condition called coronavirus disease 2019 (COVID‐19). The clinical presentation of this pathology includes fever, dry cough, fatigue, and acute respiratory distress syndrome that can lead infected patients to death. High infectivity, ability to be transmitted even during asymptomatic phase and relatively low virulence have resulted in a rapid transmission of this virus beyond geographic regions, leading to a pandemic.^1^

Since the appearance of the first cases, within a short span of just over 3 months, the infection has spread more than 200 countries across the world.^2^

To confine this potentially deadly virus, many countries all over the world and World Health Organization have strategized to interrupt the human to human contacts, isolate patients at early stages, accelerate research, and communicate correct information to the public trying to minimize the social and economic impact. There is currently a deep gap about the knowledge of the mechanisms underlying the pathogenic processes of the virus to develop specific drugs to reduce the mortality linked to the infection.

Various drugs are quickly being developed and new targets are being identified every day, and also numerous drugs are undergoing clinical trials. Interesting and discordant research have been published to provide to find the best protection of the global community before a vaccine can be made available.^3^

Coronaviruses use their S protein to enter host cells.^4^, ^5^ In humans, SARS‐2 entry happen via the host enzyme angiotensin‐converting enzyme 2 (ACE2) receptor that is located on the cells'surface.^4^, ^6^ The downregulation of ACE2 usually leads to an overproduction of angiotensin II. Angiotensin II in turn actives its 1a type receptor, and this increases the permeability of lung vessels.^4^ Therefore, the initial and the most serious prolonged injury occurs to the lungs. The immune response has a pivot role for the resolution of SARS infection, but it can also enhance, if not drive, the pathogenesis in the second phase of the disease^4^ that is is characterized by an upregulation of proinflammatory cytokines and chemokines determining a cytochinic storm in wich the interleukines‐1β (IL‐1β), 1RA, 7, 8, 9, 10, basic FGF2, granulocyte colony stimulating factor (GCSF), granulocyte‐macrophage–colony stimulating factor (GM–CSF), interferon‐γ, IP10, monocyte chemoattractant protein‐1 (MCP1), macrophage inflammatory protein 1α (MIP1α), MIP1β, platelet‐derived growth factor B, tumor necrosis factor‐α (TNF‐α), and vascular endothelial growth factor A are overproducted; some of these, like IL2, IL7, IL10, GCSF, IP10, MCP1, MIP1α, and TNF‐α could promote, or mark, disease severity.^7^ Another study shows that CD4 T cells of intensive care unit (ICU) patients with COVID‐19 produced more IL‐6 and GM–CSF than those not requiring ICU, although the numbers, powers, and treatments were not reported. The cytokine storms mediated by overproduction of proinflammatory cytokines have been observed in population with COVID‐19.^8^

Several studies are trying to discover a way to block the inflammatory process that generates the damage by using different strategies. For this reason the use of canakinumab, that is an anti‐IL‐1β human monoclonal antibody, can be considered a valid therapeutic tool for individuals with COVID‐19.^9^

We describe a first experience of hospitalized mild or severe non ICU COVID‐19 infected patients treated with canakinumab and compared with patients treated with a standard therapy (hydroxychloroquine and protease inhibitors).

## 2 MATERIALS AND METHODS

### 2.1 Design of the study

This is a single center cohort study, conducted enrolling patients admitted from March to April 2020 at the Infectious Diseases Clinic and the Internal Medicine Unit, University “G. D'Annunzio,” SS Annunziata Hospital of Chieti, Italy, with diagnosis of COVID‐19 pneumonia by real‐time PCR on oropharyngeal and nasopharyngeal swabs. Medical records related to the recovery were consulted for each patient, and data regarding anamnesis, laboratory and therapy were collected and tabulated.

### 2.2 Patient selection

All COVID‐19 patients were treated (previously or simultaneously) with standard therapy: hydroxychloroquine and protease inhibitors (lopinavir/ritonavir). All patients needed oxygen supplementation without intubation. We enrolled the first 17 patients who experienced a worsening in P/F ratio value before starting therapy with canakinumab. P/F ratio is a widely used tool to identify acute hypoxemic respiratory failure and it equals the arterial oxygen partial pressure (PaO_2_ in mmHg) from the arterial blood gas (ABG) divided by fractional inspired oxygen (FiO_2_). All the patients received the approvation for the free use of canakinumab by Novartis International AG. After getting permission from Novartis International AG we started the treatment with canakinumab consisting in a single subcutaneous administration of 300 mg. Supportive therapy was to be provided at the discretion of the clinicians. No simple‐size calculations were performed.

The standard group was made up of the first 17 patients with confirmed SARS‐CoV‐2 infection with COVID‐19 who had an oxygen saturation of 94% or less while they were breathing ambient air (FiO_2_ 21%) or who were starting having a worsening of respiratory function. The patients of the standard group did not receive any other therapy than hydroxychloroquine plus lopinavir or ritonavir to be enrolled as standard group.

### 2.3 Study assessment

Clinicians collected demographic and clinical data about the patients enrolled. Laboratory values were collected at the time of enrollment and, in the canakinumab group, before the administration of canakinumab (time 0, T0, or enrollment time), three days after enrollment (time 1, T1) and seven days after enrollment (time 2, T2). We collected data about serum creatinine, blood coagulation panel including prothrombin time time, activated partial thromboplastin time ratio, fibrinogen and *d* ‐dimer, lactate dehydrogenase (LDH), C‐reactive protein (CRP), blood urea nitrogen, troponine I, alanine aminotransferase, aspartate aminotransferase, gamma‐glutamyl transferase, alkaline phosphatase, total and fractionated bilirubin, lipase, amylase, and blood glucose levels.

Data on patients' oxygen support requirements at the three times were collected (other controls were performed at the clinician's discretion): analysis of arterial blood gases and electrolytes including P/F ratio. All the enrolled patients were studied using chest RX and/or computed tomography.

The standard group was made up of 17 patients with confirmed SARS‐CoV‐2 infection who had an oxygen saturation of 94% or less while they were breathing ambient air (FiO_2_ 21%) and who were starting having a worsening of respiratory function in standard therapy with lopinavir or ritonavir 200/50 mg 2 cps/bid and hydroxychloroquine 200 mg/bid.

The study protocol was performed in accordance with the ethical standards laid down in the 1964 Declaration of Helsinki. The patients have signed an information consent for use off‐label the drugs.

#### 2.3.1 Statistical analysis

No sample‐size calculations were performed. Quantitative variables were reported as median and interquartile range (IQR) while quantitative data were summarized as frequency and percentage. Outcomes of interest were analyzed using different linear mixed models. This approach allows explicit modeling of the within‐person and between‐person variation in the outcome, while taking into account the correlation between repeated measurements on the same individual.^10^ Linear mixed model for repeated measurements was used to regress T0, T1, and T2 measures on the fixed‐effect factors assuming unstructured covariance matrix. The p‐value were not adjusted for multiple comparisons. All the statistical analyses were performed using R software environment for statistical computing and graphics version 3.5.2 (R Foundation for Statistical Computing, Vienna, Austria. https://www.R-project.org/).

## 3 RESULTS

### 3.1 Characteristics of sample population study

Of the 34 patients enrolled with COVID‐19, 17 were male (50%), and all of them were of Caucasian ethnicity with a median age of 55.5 (IQR: 48.3–66.5) years (Table 1). No differences between the two groups were evidenced about symptoms and initial clinical conditions.

**Table 1:** Baseline clinical characteristics of patients, data are expressed as median and Interquartile range (IQR)

	Canakinumab group	Standard group	*p* Value
Gender			.368^a^
Female	2 (11.8%)	4 (23.5%)	
Male	15 (88.2%)	13 (76.5%)	
Age (year)	53.0 (48.0, 62.0)	59.0 (50.0, 72.0)	.255^b^
Clinical features at baseline			
Fever	16 (94.1%)	15 (88.2%)	.545^a^
Cough	14 (82.4%)	12 (70.6%)	.419^a^
Dyspnea	8 (47.1%)	14 (82.4%)	**.031** a
Asthenia	6 (35.3%)	3 (17.6%)	.244^a^
Nausea	5 (29.4%)	2 (11.8%)	.203^a^
Headache/dizziness	2 (11.8%)	2 (11.8%)	.998^a^
Diastolic blood pressure (mmHg)	70.0 (60.0, 80.0)	80.0 (70.0, 80.0)	.042^b^
Systolic blood pressure (mmHg)	120.0 (120.0, 130.0)	130.0 (120.0, 140.0)	.062^b^
Heart rate/min	90.0 (85.0, 99.0)	84.0 (80.0, 90.0)	.094^b^
Respiratory rate/min	20.0 (18.0, 20.0)	20.0 (18.0, 26.0)	.411^b^

*Note*: *p* < .05 are in bold.

^a^ Pearson's *χ* ^2^ test.

^b^ Mann–Whitney *U* test.

### 3.2 Ematochemical values

**Figure 1:**
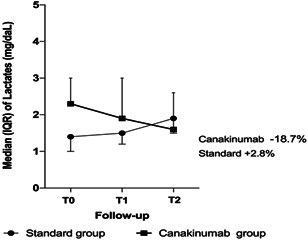
Median and interquartile range of lactates. Percentage reported in figure are the median of relative variation respect to T0 separately for two groups. Mixed linear model for repeated measure showed a not significant effect of time (*p* = .311) but a significant effect of group (*p* = .014). Interaction (time × group) result not statistically significant (*p* = .336)

With regard to the blood chemistry parameters, the patients showed a significant improvement in the number of lymphocytes (*p* = .005) and platelets (*p* < .001) over time. Also *d* ‐dimer and fibrinogen decreased significantly, with a significant reduction over time (*p* = .034 and *p* = .029, respectively). Inflammatory indices, such as CRP, have significantly normalized over time (*p* = .001), as well as LDH (*p* = .003) (Table 2). Also the lactate reduced in standard group (T0 1.4 [1.0–1.8]; T1 1.5 [1.2–2.0]; T2 1.9 [1.5–2.0]) and significally in canakinumab group (T0 2.3 [1.2–6.0]; T1 1.9 [1.0–6.0]; T2 1.6 [1.0–2.6]) (Figure 1). We have noticed an increase in liver function and renal indices but always within normal ranges (Table 2).

**Table 2:** Clinical characteristics the patients between standard group and canakinumab group at three times (T0, T1, and T2)

Variable	Baseline (T0)		Third day (T1)		Seventh day (T2)		*p* Value		
	Standard group	Canakinumab group	Standard group	Canakinumab group	Standard group	Canakinumab group	Group^a^	Time^b^	Interaction^c^
Hb (g/dl)	13.4 (13.0, 14.2)	14.6 (13.0, 15.5)	13.0 (12.2, 13.5)	13.0 (12.2, 13.5)	12.3 (11.4, 13.4)	13.5 (12.2, 14.3)	.710	**.004**	.615
WBC (cells/mcl)	7.8 (4.7, 9.0)	6.3 (4.8, 10.0)	6.4 (4.5, 7.7)	7.5 (5.3, 8.5)	6.4 (5.6, 8.0)	7.0 (5.0, 8.9)	.286	.730	.469
Neutrophils (cells/mcl)	5.8 (3.1, 7.6)	5.3 (3.7, 9.0)	4.8 (2.8, 6.0)	5.2 (4.0, 6.2)	4.5 (3.1, 5.6)	4.3 (3.6, 5.4)	.127	.230	.173
Lymphocytes (cells/mcl)	1.1 (0.8, 1.2)	0.7 (0.6, 0.9)	1.1 (0.9, 1.3)	1.1 (0.8, 1.9)	1.4 (1.1, 1.7)	1.4 (1.2, 2.0)	.146	**.005**	.074
PLT (x 10^3^cells/mcl)	213.0 (151.0, 245.0)	194.0 (174.0, 226.0)	243.0 (192.0, 326.0)	265.0 (224.0, 347.0)	299.0 (258.0, 386.0)	244.0 (208.0, 289.0)	.708	**<.001**	.065
Fibrinogen (mg/dl)	542.0 (479.0, 622.0)	639.0 (550.0, 700.0)	500.0 (450.0, 551.0)	450.0 (321.0, 530.0)	400.0 (308.5, 514.0)	360.0 (329.0, 400.0)	.869	**.029**	.166
*d* ‐dimer (ng/ml)	0.7 (0.6, 0.9)	0.8 (0.6, 1.4)	1.0 (0.9, 1.5)	0.9 (0.6, 1.1)	1.0 (0.8, 2.4)	0.5 (0.4, 0.8)	.659	**.034**	**.003**
Creatinin (mg/dl)	0.8 (0.7, 1.0)	0.9 (0.8, 1.1)	0.8 (0.7, 0.8)	0.9 (0.7, 1.0)	0.8 (0.6, 0.8)	0.8 (0.7, 0.8)	.110	.412	.241
Blood Urea (mg/dl)	35.0 (29.0, 37.0)	32.0 (24.0, 42.0)	33.0 (25.0, 38.0)	27.0 (23.0, 38.0)	28.0 (25.0, 37.0)	29.0 (23.0, 34.0)	.308	.393	.356
CRP (mg/dl)	136.3 (69.8, 197.0)	181.0 (125.3, 201.8)	140.0 (50.8, 194.7)	17.1 (2.8, 78.0)	38.0 (16.5, 76.4)	4.2 (1.5, 5.9)	.545	**.001**	.507
PCT (ng/ml)	0.1 (0.0, 0.2)	0.2 (0.1, 1.0)	0.0 (0.0, 0.1)	0.1 (0.0, 0.2)	0.0 (0.0, 0.0)	0.0 (0.0, 0.0)	**.014**	.862	.067
LDH (U/L)	300.0 (291.8, 334.0)	287.0 (245.0, 374.0)	410.0 (254.0, 490.0)	279.0 (221.0, 313.0)	226.0 (187.5, 267.0)	199.0 (184.0, 250.0)	.233	**.003**	.745
AST (U/L)	40.0 (28.0, 52.0)	29.0 (24.0, 37.0)	48.0 (23.2, 55.0)	50.0 (30.0, 109.0)	61.0 (28.5, 85.5)	58.0 (45.0, 104.0)	.233	**.003**	.745

Abbreviations: AST, aspartate aminotransferase; CRP, C‐reactive protein; Hb, hemoglobin; LDH, lactate dehydrogenase; PCT, procalcitonin; PLT, platelet count; WBC, white blodd cell.

^a^Effect of group for each variable, the differences has been tested between standard group in three times (T0, T1, and T2) and canakinumab group at three times.

^b^Effect of time, for each variable, the differences have been tested between the means of the two groups at different time.

^c^Probability that the effects of time is greater in one distinct group (interaction time × group).

### 3.3 Respiratory parameters

**Figure 2:**
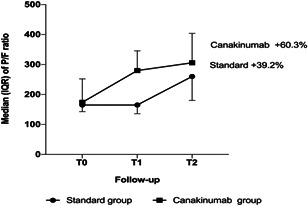
Median and interquartile range of P/F ratio. Percentage reported in figure are the median of relative variation respect to T0 separately for two groups. Mixed linear model for repeated measure showed a significant effect of time (*p* < .001) but not significant effect of group (*p* = .596). Interaction (time × group) result not statistically significant (*p* = .403)

The detailed analysis of ABG showed a faster increase of the pulmonary functions in canakinumab group in comparison to the standard group. In particular, the canakinumab group had a P/F ratio value of 174 (IQR: 138–252) at T0, a P/F ratio value of 280 (IQR: 160–345) at T1 and a P/F ratio value of 306 (IQR: 235–404) at T2. We observed a significant and rapid increase in P/F ratio in patients treated with canakinumab, in fact the canakinumab group had an improvement of the P/F ratio in 60.3% while in the standard group in 39.2% (Figure 2). At the baseline the 82.3% of canakinumab patients had an oxygen supplementation with Venturi mask at 6 L/min or more. Conversely, the 52.9% of patients in the standard group were taking an oxygen supplementation with Venturi mask at 6 L/min or more. The canakinumab group significantly reduced the supply of high oxygen flows (−28.6% at T1 vs. T0 and −40.0% at T2 vs. T1). Conversely, in the standard group increased the supply of high oxygen at T1 versus T0 (+66.7%), but reduced the supply of oxygen at T2 versus T1 (−40.0%).

Furthermore, there were no significative differences between the two groups for imaging CT or X‐ray chest at the baseline, in fact 13 patients in the canakinumab group and 14 in the standard group showed bilateral typical finding as patchy ground glass opacities.

### 3.4 Outcome e adverse events

There were no adverse events into the two groups, except for a slight increase in liver indices in the canakinumab group. No patient belonging to the two groups died. No difference in the outcome between two groups at 40 days was observed: fifteen patients were discharged without oxygen therapy in canakinumab group, while two patients of was discharged requiring oxygen therapy in standard group. Furthermore, there were no other significative differences between the two groups. No sepsis or other adverse events were detected 14 days after administration of canakinumab.

## 4 DISCUSSION

In our cohort study, canakinumab showed a significant clinical improvement of respiratory failure compared to standard therapy with hydroxychloroquine and lopinavir or ritonavir in patients infected with SARS‐CoV‐2 and mild or severe pneumonia.

Canakinumab is a human monoclonal antibody with a specific reactivity only for IL‐1 beta with no cross reactivity to other cytokines or other members of the IL‐1 family.^11^ IL‐1 is a proinflammatory cytokine capable of activating local and systemic inflammatory reactions. It can induce, acute phase plasma protein reactions with induction of the fever and bone and cartilage pain. Canakinumab, if used in juvenile idiopathic arthritis and other rare autoinflammatory conditions, such as Schnitzler syndrome, cryopyrin associated periodic syndrome and familial Mediterranean fever has been shown to improve symptoms and laboratory abnormalities.^12^, ^13^ In fact this drug was approved for use in periodic fever syndromes in the United States in 2009.^14^ Canakinumab was also tested to block inflammation in cardiovascular diseases related to a subclinical inflammation, a condition associated with an increased risk of cardiovascular events.^11^, ^15^

Several studies demonstrated that the cytokine storm, including IL1‐β, TNF‐α, IL‐12 and IL‐6 has a pivot role in the pathogenesis of severe acute respiratory syndrome associated to COVID‐19.^16^ Moreover it has been widely demonstrated that in Middle East respiratory syndrome (MERS), a disease caused by another Coronavirus called MERS coronavirus, the cytokine gene expression of different ILs, including IL‐1β, can be markedly high.^17^ Similar evidence suggests a similar pathogenesis in COVID‐19, in fact inflammatory cytokines, such as IL1‐β, are significantly elevated in patients with COVID‐19.^8^

Accordingly, inflammatory cytokine cascade blockade is a promising therapy for COVID‐19. There are some pilot studies on the use in COVID‐19 of biological drugs, such us tocilizumab (17, 18), but there is no evidence yet on the possible role of canakinumab, which acts by inhibiting the cytokine cascade at an early step. We have recently published a pilot study on safety on canakinumab in patients with mild and severe COVID‐19.^18^

In our cohort, canakinumab therapy has shown an improvement in blood chemistry parameters and in inflammation indices. This could be an important strategy to fight SARS‐CoV‐2 infection, whereas the elevated cytokine levels may be responsible of the tissue necrosis in the lung and, probably, of the lethal complications in COVID‐19 patients.^8^ The decrease of *d* ‐dimer and fibrinogen after canakinumab administration could be useful in avoiding pulmonary vascular damage, responsible of the histological changes in COVID‐19 patients. In addition, the block of the inflammatory cascade could avoid worsening patient's clinical condition. This hypothesis seems to be confirmed by the absence of worsening in the patients of our study, who instead quickly improved their pulmonary functions.

In fact, comparing patients treated with high Oxygen supports with VMK or NIV, we found that the patients in the canakinumab group, although more severely affected, reduced oxygen support quickly than the patients treated with standard therapy. Another confirmation of the patient improvement is the rapid reduction of lactate levels in patients in the canakinumab group, demonstrating the reduction of respiratory fatigue in these patients.

The present study has some limitations: it is a monocentric study, the comparator group includes patients with lower severity of COVID‐19 and only non‐intubated patients are enrolled. Finally, another important limitation is the small sample size.

## 5 CONCLUSION

In hospitalized adult patients with mild or severe non ICU COVID‐19 requiring oxygen therapy, canakinumab could be a valid therapeutic option. Canakinumab therapy was associated with a rapid and long‐lasting improvement in oxygenation levels without severe adverse events. Future randomized trials are necessary to confirm the efficacy of canakinumab in mild or severe COVID‐19 patients with respiratory failure in a larger cohort.

## CONFLICT OF INTERESTS

The authors declare that there are no conflict of interests.

## AUTHOR CONTRIBUTIONS

Falasca Katia, Di Penta Myriam, and Claudio Ucciferri were responsible for the conception, design and write of this study. Marta Di Nicola and Michele Marchioni performed analysis and interpretation of all the data. Antonio Auricchio, Celletti Eleonora, and Sabatini Emanuela: data collection. Francesco Cipollone and Jacopo Vecchiet were revisioned the manuscript. All authors read and approved the final manuscript.

## Data Availability

The data that support the findings of this study are available from the corresponding author upon reasonable request.
